# Total Sugar Intake and Macro and Micronutrients in Children Aged 6–8 Years: The ANIVA Study

**DOI:** 10.3390/nu12020349

**Published:** 2020-01-29

**Authors:** María Morales-Suarez-Varela, Isabel Peraita-Costa, Agustín Llopis-Morales, Yolanda Picó, Maira Bes-Rastrollo, Agustín Llopis-Gonzalez

**Affiliations:** 1 Unit of Public Health and Environmental Care, Department of Preventive Medicine and Public Health, Food Sciences, Toxicology and Forensic Medicine, School of Pharmacy, University of Valencia, Av. Vicente Andrés Estellés s/n, 46100 Burjassot, Valencia, Spain; ivperaitacosta@hotmail.es (I.P.-C.); agustinllopis@gmail.com (A.L.-M.); agustin.llopis@uv.es (A.L.-G.); 2 CIBER of Epidemiology and Public Health (CIBERESP), Instituto de Salud Carlos III, Av. Monforte de Lemos, 3-5. Pabellón 11. Planta 0 28029 Madrid, Madrid, Spain; Yolanda.Pico@uv.es; 3 Environmental and Food Safety Research Group (SAMA-UV), School of Pharmacy, University of Valencia, Av. Vicent Andrés Estellés s/n, 46100 Burjassot, Valencia, Spain; 4 Research Center on Desertification (CIDE, UV-CSIC-GV), Carretera Moncada-Náquera, Km 4,5 46113 Moncada, Valencia, Spain; 5 Navarra’s Health Research Institute (IdiSNA), C/Irunlarrea 3, 31008 Pamplona, Navarra, Spain; mbes@unav.es; 6 CIBER of Physiophatology of Obesity and Nutrition (CIBERobn), Instituto de Salud Carlos III, Av. Monforte de Lemos, 3-5. Pabellón 11. Planta 0 28029 Madrid, Madrid, Spain

**Keywords:** sugar intake, children, eating patterns, body composition, public health nutrition

## Abstract

The objective of this study was to study the association between total sugar intake (TSI) levels of children aged 6–8 years old, nutrient intake and anthropometry. Food and beverage intakes were collected by a prospective three-day recall questionnaire. The 2237 children were distributed into three groups according to TSI percentiles. Mean TSI was 93.77 ± 25.72 g/day, 22%–25% of total caloric intake, with boys presenting an intake of 96.24 ± 24.34 g/day and girls 91.38 ± 26.78 g/day. Greater TSI was associated with higher body fat, parental education, energy intake, nutrients/1000 kcal, and lower weight z-scores, BMI z-scores, waist circumferences, and hip circumferences. Weight, height, and waist circumference had the highest *R*^2^ while body fat had the lowest. The percentage of total energy derived (%E) from protein decreased as the %E from TSI increased, while the opposite was true for carbohydrates and saccharides, while for fiber intake, the medium groups presented the highest intake/1000 kcal. For the remaining macronutrients studied, intake/1000 kcal decreased when the %E from TSI increased. Calcium, iodine, magnesium, vitamin B2, folate, and vitamin C intake increased as the %E from TSI increased, while the opposite was true for vitamin B12. Fiber, ω-6 PUFA, iodine, folate, vitamin D, and vitamin E intakes were insufficient across most of the sample. TSI levels in children were identified to exceed adult recommendations. It is not clear what the effect of up to an average of 21% of energy coming from total sugars has on childhood obesity and further research is needed in the pediatric population, however, opportunities exist to improve sugar intake patterns.

## 1. Introduction 

The main function of sugar is to provide the body with energy after breaking down into glucose, but from a nutritional perspective, sugar is not an essential nutrient, as glucose may be produced by the body from fat and protein [1]. However, a healthy diet contains at least some amount of naturally occurring sugars, because these are integral parts of fruit, vegetables, dairy, and grains [2]. Dietary sugar intake has gained prominence as a highly controversial subject in regard to public health [3,4,5]. Children are exposed to an ever-increasing number of convenience and fast foods high in fat and sugar and are vulnerable to their appeal which may contribute to the increased prevalence of overweight and obesity among children [6]. 

Sugar has several functional properties in food and currently no other sweetener can duplicate all or even many of them [7]. These properties are derived from the sensory and physical properties of sugar and its reactions and interactions with the other ingredients [8]. The most notable function of sugar in food is its sweet taste which serves as a sensory cue for energy as well as a source of pleasure. Sweetness is an innate taste, and it has been argued that a preference for sweetness evolved to ensure that animals and humans chose non-toxic foods high in calories [8]. Sweetness also improves the palatability of food thus increasing the probability that they will be consumed. Sugar contributes to the flavor profile of a food by interacting with other ingredients to enhance or lessen certain flavors. Sugar can also affect the physical properties of food, such as volume and texture, to a significant degree, and sometimes specific sugars are used as bulking or texturizing agents or as part of the fermentation process [8,9]. The hygroscopic nature of sugar plays a crucial role in food preservation, as it reduces water activity in foods making it unavailable for chemical or biochemical reactions [8,9,10]. 

Although conflicting studies demonstrate some uncertainty on this topic, it is plausible that sugar consumption may be a factor that has driven changes in obesity rates [11]. While some studies have found that sugar intake is associated with the risk of developing some diet-related chronic diseases, such as obesity, metabolic syndrome, diabetes, cardiovascular and musculoskeletal diseases, dental caries, and hyperactivity [12,13,14,15,16], recent systematic reviews and meta-analysis have shown that sugar per se does not appear to increase body weight nor is it associated with diet-related diseases, except for dental caries, under isocaloric conditions [17,18,19,20]. Other than energy intake, energy expenditure constitutes the most important factor in the energy balance formula, and physical activity is the only modifiable variable that affects energy expenditure [11,21]. Although energy intakes have not increased significantly, there has been a decrease in physical activity which may result in significant changes in body weight and/or composition [6].

In this study, the term “sugar” has been used to refer to total sugar which is defined as all monosaccharides and disaccharides added to foods and beverages by the manufacturer, cook or consumer and, also, sugars naturally present in honey, syrups, fruit juices, fruit concentrates as well as in milk, fruit, and vegetables [22]. Currently there is no official pediatric sugar intake recommendation, the only available recommendation for sugar is the adult reference intake which is established at a maximum of 90 g per day [23]. Considering the lack of official reference intakes for the pediatric population, the Institute of Grocery Distribution proposed a reference intake of 85 g per day for children under 18 years old [24] which was rejected by the UK Department of Health [25]; therefore, no official pediatric reference intakes are available for comparison. The formulas for daily energy requirements prepared by the Federación Española de Sociedades de Nutrición, Alimentación y Dietética (FESNAD) in 2010 [26] yield a recommended daily caloric intake of between 1500 and 1700 kcal for the Spanish population of 6 to 8 years old depending on age, height, weight, and physical activity level. Taking these values, the proposed reference intake of 90 g of sugar would represent a little above 20% of total daily caloric intake.

Regarding the provenance of this sugar, the World Health Organization (WHO) and the Spanish Society of Community Nutrition (SENC) recommend that the population average intake of free sugar (monosaccharides and disaccharides added to foods and beverages by the manufacturer, cook or consumer, and sugars naturally present in honey, syrups, fruit juices, and fruit juice concentrates) should not exceed 10% of total caloric intake and also advise that consumption is optional and should be occasional, considering that a reduction to below 5% would produce additional benefits for health [1,27]. The basis for this goal is that high intakes of sugars are associated with decreased nutrient density and risk of weight gain, especially when consumed as beverages [16]. 

Childhood is an important period in human life in which implementing good lifestyle habits is essential. Early childhood is the most rapid period of development and following a balanced diet at this stage is essential to ensure optimal growth and development [28]. Currently, 60% of Spanish population suffer from being overweight or obese, being this especially worrying in children because it affects 8% of girls and 13% of boys [29]. This places Spain as the second country in Europe with more obese people [29]. This information highlights the importance of monitoring nutritional status and implementing health education programs in children [28]. Sugar intake is not an isolated component of the diet; therefore, it needs to be evaluated along with the total dietary pattern (macro and micronutrients). Dietary surveys provide insight into dietary habits and food intake and help estimate the adequacy of nutrient intake of different groups in a population [28]. 

Very little data are available regarding the total intake of sugars in children, their relationship with macro and micronutrients from diet and their association with anthropometric data and no data exist for Spanish population of this age. A meta-analysis has confirmed the relation between sugar intake and anthropometric measures [17]. The effect of dietary sugar intake on weight appears to result from the extent to which increasing or decreasing intakes in free-living individuals influence energy intakes, because no change in weight is apparent when proportions of total energy derived from sugar are altered in the context of strict energy balance [30]. Living in a world where the prevalence of obesity is reaching historic highs [11,31], especially in children, and having reviewed literature about sugars intake, information about the relationship between them and nutrition/anthropometric variables/demographic characteristics is relatively scarce. Several theories have attempted to explain the rising levels of obesity, and altered patterns of sugar consumption may play a role, as changes in sugar intake levels have been shown to occur simultaneously to changes in obesity rates [11]. 

The objective of the present study was to identify total sugar intake and its association with macro and micronutrient intake along with anthropometric measures in Valencian children aged 6 to 8 years old.

## 2. Materials and Methods

This study is part of a larger study (ANIVA) centered on the dietary pattern and habits of the sample population whose results are compiled and derive into nutritional intervention when required [32]. The study protocol was reviewed and approved by the University of Valencia Institutional Review Board Ethics Committee (2014/29630). It complies with the Declaration of Helsinki Guidelines, was approved by the Autonomous Secretariat of Education, Generalitat Valenciana, Valencia (Spain), and written consent was obtained from all children’s parents/guardians.

### 2.1. Population and Sample

This cross-sectional study used a sample of 2237 schoolchildren, of which 1098 were boys (49.1%) and 1139 girls (50.9%), between 6 and 8 years of age belonging to the Valencian Community (Spain) and part of the ANIVA (Anthropometry and Child Nutrition of Valencia) Study [32].

Data collection began with a presentation to the schools of a formal introductory letter accompanied by the authorization of the project by the Autonomous Secretariat of Education and the University of Valencia. Subsequently, a meeting was arranged with the school director and parent’s association to present the study. After acceptance by both aforementioned parties, the school was considered a participating center.

**Figure 1:**
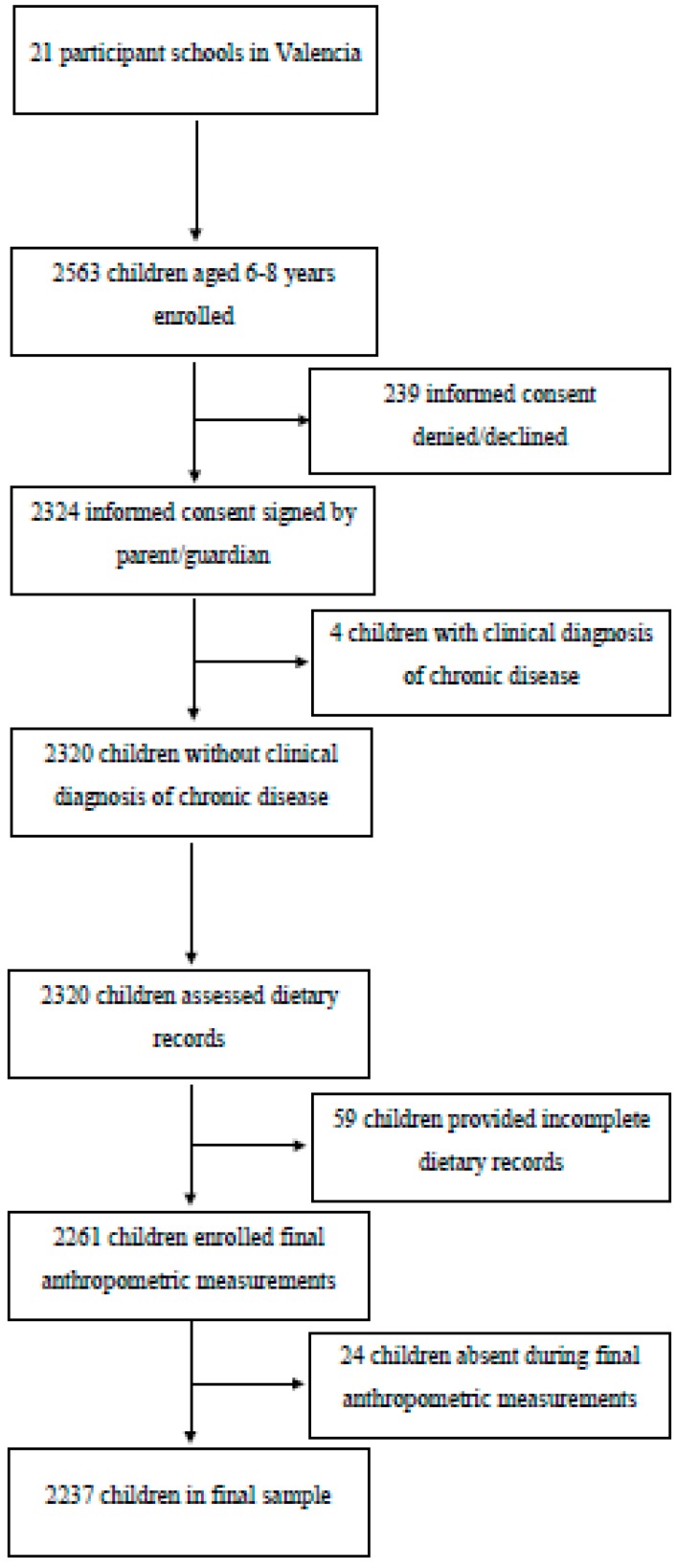
Recruitment of children for participation.

A total of 21 schools participated in the study. The schools were randomly selected within the Valencian Community and all schools contacted agreed to participate. Two thousand two hundred and fifty-six children were initially recruited, and a letter was sent to their parents or guardians with the informed consent attached. For 2324 children, the parents/guardians gave informed consent to participate in the study of which 4 had to be excluded due to the fact of a clinical diagnosis of a chronic illness affecting their diet. After consent was returned, the survey to be completed by parents or guardians was given to the 2320 eligible children. This survey consisted of a description of the study, where it was explicitly stated that the collected data would remain confidential in accordance with the Spanish Personal Data Protection Act, instructions for completion and a brief questionnaire aimed at knowing the level of studies of the parents or guardians and the physical activity of the student, as well as a record of dietary intake. A total of 2262 children returned complete food journals and questionnaires. Of these children, 24 were absent during the day designated for the taking of anthropometric measurements leaving a final sample of 2237 children. The recruitment and selection process is detailed in the flowchart presented in Figure 1.

### 2.2. Anthropometric Measures

The anthropometric measurements taken during the health examination were taken following the standard procedures described by the WHO [33], with children barefoot and wearing light clothing. Anthropometric measurements were taken twice and averaged. The anthropometric data collected were the weight and body fat percentage, using a Soehnle Body Balance Comfort Select 63760 calibrated electronic digital scale with a capacity of 150 kg and a precision of 0.1 kg, the height, through a Seca 213 portable stadiometer that allows measuring up to 250 cm with division in millimeters and waist–hip circumference through a tape measure. The waist/hip index was calculated by dividing the waist measure by hip measure for each individual.

Once the values obtained for each individual were recorded, the WHO Anthro software version 3.2.2 of January 2011 (World Health Department of Nutrition, Geneva, Switzerland) was used to calculate body mass index (BMI) for age (z-score), weight for age (z-score), and height for age (z-score) in order to obtain a comparison between the real values obtained in the measurement of each child and those growth values that would be optimal. The standard deviation values used in the study are governed by the WHO International Child Growth Standards [33].

### 2.3. Diet Assessment

The analysis of the dietary intake was carried out through a three day, one of which was a weekend/holiday, food journal previously validated for a pediatric population. The questionnaire used was specifically designed to be used with the DIAL software for diet assessment and food calculations (Department of Nutrition (UCM) & Alce Ingeniería, S.L. Madrid, Madrid, Spain) which provides a detailed assessment of the diet by generating macro and micronutrient data corresponding to each individual which would be included in a database for later statistical analysis [34]. The software has been previously validated and the food database was last updated in August 2018. The database has a nutritional composition table which contains vast information on the composition in terms of energy, proteins, lipids, carbohydrates, fiber, minerals, vitamins, cholesterol, fatty acids, amino acids, etc. (up to a total of about 140 different components), for more than 800 of the most common foods. The information of a product can be located not only by the most common name but also by using local, regional or national names along with the possibility of searching using the scientific name and its equivalence in English. The software also uses a table of homemade measurements, with about 1860 entries, with the units and rations most commonly used. Therefore, nutrient intake is estimated using both the type of food consumed and the quantity. The software also allows to estimate the recommended intakes of energy and nutrients according to the individual characteristics of a person: age, sex, weight, and physical activity. 

The tables for the Spanish population of 6 to 8 years old prepared by FESNAD in 2010 were used for recommended daily intake (RDI) comparison when available [26]. The RDI is the average daily dietary intake level that is sufficient to meet the nutrient requirements of nearly all (97–98 percent) healthy individuals in a particular life stage and gender group. 

Adherence to the Mediterranean diet was measured using the KIDMED questionnaire [35] which was developed to quickly and easily assess the degree of adherence to the Mediterranean diet which, given the characteristics of the study population, should be the most prevalent dietary pattern [35]. The KIDMED test has been successfully used in numerous previous studies [36,37,38,39,40,41]. The degree of adherence to the Mediterranean diet was assessed based on intake of dietary compounds positively and negatively associated with the Mediterranean diet. Products positively associated (i.e., vegetables, legumes, fruits, nuts, cereal, fish, dairy products, oil) were assigned a value of +1, whereas products negatively associated (i.e., sweets, fast foods) were assigned a value of −1. Adherence was split into 3 levels: ≥8 optimum; 4–7 average; and ≤3 low.

### 2.4. Physical Activity

To assess children’s physical activity, parents were asked to report the number of days a week that their children practiced physical activity and the duration of these sessions in minutes. The questionnaire used was based on the National Health Survey 2011–2012 conducted by the Ministry of Health, Social Services, and Equality of Spain [42]. Physical activity was classified at two levels: adequate (at least 60 min of moderate to vigorous physical activity a day) and inadequate (less than 60 min of moderate to vigorous physical activity a day) [43]. In addition, parents were asked to report their children’s weekly frequency of sedentary activity, such as the hours spent watching TV, on the computer, and playing video games. 

### 2.5. Socio-Educational Level

The child’s environment was assessed through questions about the education of the parent/guardian which was classified into: without studies; primary studies, secondary studies; or university studies and postgraduate university studies.

### 2.6. Statistical Analysis

Data checking was performed both manually and statistically with outliers for the measured and derived variables identified by z-scores and with cross-checking against the original data collection sheets.

Descriptive statistics were calculated for anthropometric measures, and the macro and micronutrients, and the results were expressed as means ± standard deviations (SDs). Normality of the distribution of continuous variables was assessed by the Kolmogorov–Smirnov test. The quartiles of sugar intake for boys and girls were calculated. 

The sample was stratified according to sex-specific quartiles of either or both total sugar intake and percentage of total energy derived from total sugar intake. The 1st quartile was defined as a low sugar consumption group (P25), the 2nd and 3rd quartiles as the medium sugar consumption group (P25–P75), and 4th quartile as the high sugar consumption group (P75). Differences between P25, P25–P75, and P75 for all studied variables are presented in the tables. 

The possible association between anthropometric variables, macro and micronutrients in relation with sugar intake was analyzed with a one-way ANOVA Tukey’s post-hoc test, as all variables were normally distributed. 

In the specific case of the possible association between anthropometric variables and the percentage of total energy derived from total sugar intake, a multiple regression analysis adjusted for age and physical activity after stratification according to sex-specific quartiles of percentage of total energy derived from total sugar intake was performed to obtain *R*^2^ coefficients. 

For all analyses, values of *p* < 0.05 were considered to indicate statistical significance (two-tailed test). The statistical analysis was performed using IBM SPSS Statistics v.24 for Windows.

## 3. Results

The study included 2237 children: 1098 boys and 1139 girls (*p* = 0.371). 

The total sugar intake consumed by the studied 6–8 years old children is presented in Table 1, while sugar intake breakdown stratified by gender is shown in Table 2. Mean total sugar intake WAS 93.77 ± 25.72 g/day (22% to 25% of total energy intake) with boys presenting an intake of 96.24 ± 24.34 g/day (23% to 26% of total energy intake) and girls 91.38 ± 26.78 g/day (22% to 24% of total energy intake). Median intakes were 91.85 g/day (22% to 24% of total energy intake) for the total sample with 94.90 g/day (22% to 25% of total energy intake) for boys and 88.80 g/day (21% to 24% of total energy intake) for girls. Differences between boys and girls for total sugar intake (*p* < 0.001), glucose (*p* < 0.001), fructose (*p* < 0.001), sucrose (*p* = 0.036) and lactose (*p* = 0.021) were significant, but no differences were found for galactose (*p* = 1.000) or maltose (*p* = 0.344). It was identified that the sugar intake of more than 50% of children who participated in the study was above the recommended adult daily intake of 90 g.

Based on dietary intake of sugar stratified by sex-specific percentiles, 276 boys (12.3%) were grouped in the P25 (<79.9 g/day), 542 were grouped in the P25-P75 (79.9–112.0 g/day) with P50 being an intake of 94.9 g/day of sugar and 267 (11.9%) were grouped in the P75 (>112.0 g/day); at the same time, 324 girls (14.5%) were grouped in P25 (<74.5 g/day), 526 were grouped in the P25-P75 (74.5–106.0 g/day) with P50 being an intake of 88.8 g/day of sugar and 284 (12.7%) in P75 (>106.0 g/day). 

Table 3 shows some stratified sociodemographic characteristics such as parent’s level of education, physical activity, and adherence to the Mediterranean diet score. Significant differences were found regarding the parental education level for both genders. No significant differences were found for level physical activity and only in the group of girls were significant differences in Mediterranean diet adherence found. Adherence in girls with high sugar intakes was worse than in those with lower intakes.

Table 4 shows some stratified sociodemographic characteristics, such as parent’s level of education, physical activity, and adherence to the Mediterranean diet score, in relation to % of total energy intake from sugar intake. No significant differences were found regarding the parental education level, physical activity or in Mediterranean diet adherence for any of the groups.

Table 5 summarizes the mean values of age, weight, height, BMI, waist and hip circumferences, waist/hip index, and % fat according to gender and the amount of sugar intake. Significant differences were observed in age (*p* = 0.026), weight z-score (*p* = 0.003), height z-score (*p* = 0.030), BMI z-score (*p* = 0.013), hip circumference (*p* = 0.041), and % fat (*p* = 0.024) in the boys group, whereas significant differences were observed in weight z-score (*p* = 0.018), BMI (*p* = 0.042), BMI z-score (*p* = 0.007), waist circumference (*p* = 0.002), and % fat (*p* = 0.001) in the group of girls.

Table 6 shows the mean anthropometric values of the children studied in relation to sex-specific groups of total sugar intake adjusted for total energy intake. Significant differences were observed in weight z-score (*p* = 0.049), waist circumference (*p* = 0.010), and % fat (*p* < 0.001) in the boys group, whereas significant differences were observed in weigh (*p* = 0.050), weight z-score (*p* = 0.020), height (*p* = 0.005), height z-score (*p* = 0.038), BMI z-score (p = 0.014), waist circumference (*p* = 0.009), hip circumference (*p* = 0.003), and % fat (*p* < 0.001) in the group of girls. No significant differences were found for BMI or waist/hip index. 

Table 7 shows the results (*R*^2^ coefficient of determination) of the multiple regression analysis adjusted for age and physical activity after stratification according to sex-specific quartiles of percentage of total energy derived from total sugar intake for the possible association between anthropometric variables and the percentage of total energy derived from total sugar intake. While the *R*^2^ does not indicate whether the independent variables are a cause of the changes in the dependent variable, it does permits us to know how well belonging to a specific sugar intake group predicts anthropometric values. The highest R^2^ values (~0.3) were found for weight and height with the corresponding *p*-values all ˂0.05. This would mean that the model explains ~30% of the variation within the data and also indicates statistical significance. Body mass index also presents statistical significance for all groups while waist and hip are significant in all groups except the boys P25. Waist/hip index is only statistically significant in boys P25, boys P25-P75, and girls P25-P75. The *R*^2^ values for the z-scores are low, between 0.001 and 0.113, and statistical significance was only found in the boys P75 group for weight z-score and BMI z-score. Meanwhile, fat percentage presented the lowest *R*^2^ values (0.001–0.006) and no statistical significance was found in any of the groups.

Table 8 shows macronutrient intake according to gender and sugars intake. For both groups, boys and girls, on average, presented higher intakes of total energy, protein, carbohydrates, monosaccharides (glucose, fructose and galactose), disaccharides (lactose, maltose and sucrose), fiber, cholesterol, and lipids when sugar intake was higher. The intake of fatty acids was significantly higher among children with higher sugar intakes for SFA, myristic acid, palmitic acid, stearic acid, MUFA, palmitoleic, oleic acid, PUFA, ω-6 PUFA, linoleic acid, linolenic acid but for ω-3 PUFA the boys P25-P75 group presented the highest intake values. 

Table 9 shows the intake of macronutrients stratified by sex-specific quartiles of percentage of total energy derived from total sugar intake. The percentage of total energy derived from protein decreases as the percentage of total energy derived from total sugar intake increases while the opposite is true for carbohydrates, glucose, fructose, galactose, lactose, maltose and sucrose. For fiber intake, the P25-P75 groups present the highest intake per 1000 kcal. For the remaining macronutrients studied, intake per 1000 kcal decreases when the percentage of total energy derived from total sugar intake increases. Myristic acid is the only nutrient that does not present statistically significant differences among the groups.

Table 10 shows the intake of micronutrients according to gender and sugar intake. There was a higher intake of micronutrients among children with P75 versus P25, in all studied micronutrients that presented statistical significance (*p* < 0.05). No differences in the intake of vitamin B1 and folate in either gender were observed.

Table 11 shows the intake of micronutrients per 1000 kcal stratified by sex-specific quartiles of percentage of total energy derived from total sugar intake. The intake of calcium, iodine, magnesium, vitamin B2 and vitamin C increased as the percentage of total energy derived from total sugar intake increased. For iron and vitamin B6 intake, the P25-P75 groups presented the highest intakes. No differences in the intakes of zinc, vitamin A, vitamin B1, vitamin D or vitamin E in either gender were observed. If stratified by sex, in boys, the intake of folate and vitamin B12 increases as the percentage of total energy derived from total sugar intake increases and, in girls, this was also true for folate.

Comparing the intake of macro with the RDI, an intake higher than recommended for energy was observed in all groups with the intake of protein quadrupling the RDI and carbohydrate intake 1.5 to 2 times higher than the RDI. Fiber and ω-6 PUFA intake were lower than recommended in all groups for both genders while linoleic acid intake was lower than the RDI in the P25 group of both boys and girls. Iodine intake requirements were only met by the P75 group in boys. Insufficient folate and vitamin D intakes were observed in all groups studied with the results for folate being of particular interest as the highest intakes found, in the P75 groups, representing less than 10% of the RDI. Meanwhile, vitamin E intake was lower than the RDI in the P25 group of boys and the P25 and P75 groups of girls. 

## 4. Discussion 

This study provided total sugar intake data for Valencian children (Spanish) where the mean intake was 93.77 ± 25.72 g/day (22% to 25% of total energy intake) and the median 91.85 g/day (22% to 24% of total energy intake). It was identified that sugars intake by more than 50% of children who participated in the study was above the EU recommendations of 90 g/day or 20% of total energy for adults. Given the body mass of children of the age studied compared to the average adult, for which these recommendations were established, it would be logical that the optimal total sugar intake for a population 4–8 years old would be quite lower, meaning that an even higher percentage of children would exceed recommendations. High total sugar intake was associated with higher fat percentage and unbalanced macro and micronutrients intakes. The result of high sugar intake being associated with higher body fat percentage could be interpreted as excessive total sugar intake being responsible, at least in part, for metabolically obese but normal weight children who present an inadequate dietary pattern.

The mean sugar consumption of 93.77 ± 25.72 g/day in children 6–8 years old was higher than that described in the ANIBES study, the goal of which was to evaluate energy intake and energy expenditure in a nationally representative sample of the Spanish population 9–75 years old and which identified a mean total sugar intake of 91.6 g/day for the 9–12 years old age group [44,45,46]. As in this study, differences in terms of gender were found with the ANIBES study identifying a higher consumption of sugar in males compared to females [44,45,46]. A review of the sugar consumption from nationally representative surveys across the world [47] found that for the 15 studies conducted on children between 4 and 10 years of age and which had information on total sugar consumption, total sugar consumption varied from 83.6 g/day to 167 g/day, and that the percentage of total energy that total sugar represented was from 17% to 34.8%. In all the reviewed studies, boys presented higher total sugar intakes than girls. The results from this study would therefore be in line with the findings of this review for total sugar intake, percentage of total energy, and gender disparity in sugar intakes. The European EDIFIC study found that total sugar intake in 2–9 years olds varied from 77 g/day (19% total energy) to 114 g/day (30%) [48]. A Canadian study found that children 4–8 years old consumed 120 g/day of total sugar equivalent to 26% of total energy [49]. A Portuguese study on 5–9 years olds found mean total sugar intake to be 100.0 g/day and 22.7% of total energy [50]. Meanwhile, a Dutch study found a median intake of 135 g/day, representing 28% of total energy [51], and a mean of 143.1 g/day or 25.8% of total energy in children 7 and 8 years old [52] with boys once again having higher sugar intake values. Two studies using information on French children found mean sugar intakes of 93.6 g/day or 20.7% of total energy [52] and 98.6 g/day or 22.2% of total energy [47]. It would seem that our results for total sugar intake fall in line with those previously found by other studies in similar age groups and the finding that boys have higher total sugar intakes are also in accordance with the aforementioned studies.

In this study, it was identified that total energy intake exceeded the RDI in all groups which was also seen in previous studies in similar age groups [47] but not in others [53,54], and this excess caloric intake is associated with higher levels of total sugar intake [47]. In general, there was a higher consumption of macronutrients in the P75 group, but no group met the RDIs for fiber and ω-6 PUFA. It must be noted that carbohydrate intake exceeded recommendations in all studied groups, and the reduction of its intake might be beneficial in aiding to achieve a more balanced diet. Regarding the intake of micronutrients, fundamental in the studied age group for proper growth and development, there were some significant deficiencies. The sample was noticeably deficient in folate with the groups with the highest intakes only reaching about 8% of the daily recommendation. In infants and children, folate deficiency can lead to failure to thrive or slow growth rate, an abnormally small head, megaloblastic anemia, diarrhea, oral ulcers, neurological deterioration, irritability, developmental delay, seizures, blindness, and cerebellar ataxia [55]. Recommended intake for iodine was only met by the P75 groups, and intakes of vitamin E were borderline with the RDI in the P25 groups. When comparing the results obtained with the ENALIA [56] survey, the current study detected a lower intake in at least half of the studied groups. Given the results in dietary intakes that appear in this study, the negative association between high sugar intake and lower dietary intakes of micronutrients must be reconsidered, as it appears that the it could be related mainly with inadequate eating patterns and not with total sugar intake per se, although, a high consumption of sugars may contribute to an inadequate dietary model [57,58]. If the majority of total sugars came from healthy food sources such as fruits, vegetables or dairy products, the nutrient deficiencies identified in this study should not have been detected, as the intake of sugar would be accompanied by the intake of the necessary nutrients. This leads us to think that the sugars mainly consumed by the children in this study were added sugars, and any negative association between dietary sugar and diet quality is better exposed by referring to added sugar rather than total sugar [59]. 

Some studies, such Malik et al. [60] and Stern et al. [61], observed a positive association between sugar intake and weight gain and waist circumference [60,61]. However, in this study, anthropometrically speaking, the only differences found between the P25 and P75 total sugar intake groups were in hip circumference and body fat percentage in boys and BMI and waist circumference and body fat percentage in girls. However, when comparing the P25 and P75 groups according to the percentage of total energy from sugar, differences were found in weight z-score, height z-score, waist circumference and % fat in boys and weight, weight z-score, height, height z-score, BMI z-score, waist circumference, hip circumference, and % fat in girls. For all values except for % fat, the P75 group presented lower values than the P25 group, meaning that as the percentage of calories derived from sugar in a child’s diet increased, their anthropometric values decreased. The opposite was found for % fat, however, with those consuming a higher percentage of calories from sugar having significantly higher body fat percentages: 19.02 ± 6.78 for boys and 21.59 ± 6.20 for girls. 

Body fat percentage was selected as an outcome in view of the extent to which comorbidities of obesity contribute to the global burden of non-communicable disease [16]. This identified increase in fat percentage among boys and girls with high sugar intake is consistent with some previous studies [62,63]. A previous meta-analysis shows a relation between sugar intake and body weight but none between sugar intake and adiposity in kids [17], which is exactly the opposite of what is observed in this study. One of the explanations presented for the association between sugar intake and body weight is the extent to which sugar intake influences total energy intake [17,30] which is also reflected in this study, as a higher sugar intake is associated with a higher total energy intake. However, in this study, the higher total energy intake of the P75 groups is accompanied by an increase in the percentage of total intake that sugars accounts for—around a 5% increase. An increase in the proportion of sugar consumed could mean that the children in the P75 groups are replacing healthier food options with foods high in sugars and calories which in turn may explain the association between adiposity and sugar intake. This could also explain why Mediterranean diet adherence in girls with high sugar intakes was worse than in those with lower intakes; these girls may be consuming a higher quantity of empty calories without nutritional value. 

Previously, increases in the proportion of energy intake derived from sugars have not been associated with weight changes [30]. However, it is known that isocaloric diets differing in macronutrient composition may result in preferential partitioning of energy storage toward body fat and may over the long term alter the proportions of body fat and fat-free mass [64]. A previous study found that there was no difference in the source of calories when it comes to body fat differences among controlled isocaloric diets varying in the ratio of carbohydrate to fat [64]. Nevertheless, it is possible that isocaloric diets differing in carbohydrate and fat may have overall health effects unrelated to total body fat such as carbohydrates playing a role in determining the location of body fat stores [65,66,67]. A more in-depth analysis of the sources of the total calories consumed by the studied children may help to shed more light on the association between dietary intakes and anthropometric values.

Although the association identified of sugar intake on anthropometric measures was relatively modest, a reduction of intake is likely to have public health relevance, especially in the context of the modification of several risk factors that have synergistic effects in terms of cardiovascular risk [68,69,70]. A previous meta-analysis concluded that total sugar was not associated with cardiovascular disease incidence in extreme quantile analyses or in linear and nonlinear dose-response models but a harmful association with cardiovascular disease mortality was seen in nonlinear dose-response models with a threshold for harm above intakes of 133 g/day (26% total energy intake) [70]. Although an understanding of the extent to which changes in energy intake and body weight influence the effect of sugar on cardiovascular risk is of inherent interest, the individual and public health benefits that might be expected to arise from the reduction in intake of sugar should not be dependent on the previous understanding of this mechanism [30].

The current study identified that a high level of parental education is associated with the high consumption of sugars which is contrary to other previously published studies in which a higher level of parental education is associated with healthier eating patterns [71,72,73]. However, in developing countries, childhood obesity is most prevalent in the wealthier sections of the population [74], while a review of studies on adults found relative body size to be greater among those with lower income, particularly among women [75]. 

Parental education levels are often used as a measure of household socioeconomic status as well as the family income. Subjects with higher education have more income to spend on food and are more health conscious. They will not only be more likely and able to adopt much healthier dietary habits themselves, but this will also affect their choices regarding their children’s diet. Foods high in added sugars are less costly than foods with high nutrient density [76,77] and added sugars intake is directly related to the amount of food money available [78]. The low cost and high palatability of added sugars and fats may explain why low-income families have higher rates of overweight and obesity [76,79,80].

Over the past few decades, food and home environments have changed tremendously and there are more families in which both parents work, and time constraints have become a crucial factor in determining the types of foods consumed [6]. The results of our study emphasize the need for nutrition education programs for parents. 

### Strengths and Limitations

One of the limitations of this study was that the assessment of dietary data was challenging, especially given the complexity of the relationships between the different macro and micronutrients in this critical stage of life and the lack of appropriate reference values for comparison. Methodological differences may account for different findings. Sugar intake in many studies is assessed using a questionnaire of added sugar, where in the current study, a dietary questionnaire of three days was used to calculate total sugar intake. This study was observational, and the findings should be ideally proofed by blood analysis.

To focus solely on total sugar intake may be a too narrow view on this topic given that when evaluating the risk associated with a single food group, one always must consider that there are interactions among food groups. If these interactions are not appropriately identified or accounted for, they may influence the results and conclusions derived from them. 

Another limitation of this study was that sugar intake and data on potential confounders were only assessed at one moment in time and not during a follow-up. Therefore, there exists the possibility of under or overreporting in regard to the adequacy of micronutrient intake. 

The strength of this community-based study is its large sample size with a prospective food frequency questionnaire of three days with complete information of all intake for each child. Actual food consumption at the individual level was estimated to provide summary data at the subpopulation or population level for children in Spain. 

## 5. Conclusions 

The mean total sugar intake of the sample was 93.77 ± 25.72 g/day with boys presenting an intake of 96.24 ± 24.34 g/day and girls 91.38 ± 26.78 g/day and all being above the established reference intake for adults which should lead to a reflection on the adequacy of the diets of the children studied and the necessity to reduce sugar intake to improve overall health perspectives. The anthropometric values of girls with high sugars intake related to lower values in terms of weight z-score, BMI z-score, and waist circumference and higher values of percentage of body fat, while boys with high sugars presented lower values for weight z-score and height z-score and higher values for percentage of body fat. Overall, in this population of children, higher sugar intake was associated with lower weight z-score, lower BMI z-score, lower waist circumference, and lower hip circumference. However, conversely, it was found that higher sugar intake was also associated with significantly higher body fat. The findings support the idea that reducing sugar intakes might be expected to reduce the percentage of fat in the body therefore reducing or even eliminating it as a risk factor for diet-related chronic diseases. 

Parents whose children consumed more sugar had a higher level of education. Mediterranean diet adherence in girls with high sugar intakes was worse than in those with lower intakes. 

In dietary terms, children with the highest sugar intake consumed more calories; however, all children were consuming well beyond the recommendations for total energy and, thus, for macronutrients, yet were not achieving the recommended fiber intakes. For micronutrient intake, no consistent pattern associated with sugar intake was observed. When compared with available macro and micronutrient RDIs, fiber, ω-6 PUFA, iodine, folate, vitamin D, and vitamin E intakes were insufficient across the majority of the sample. 

In conclusion, it is not clear based on these results what the effect of up to an average of 21% of energy coming from total sugars has on childhood obesity and further research is needed in the pediatric population. However, a nutritional intervention to lower total sugar intake in children may have the potential to reduce body fat percentage leading to a decreased risk for multiple comorbidities associated with overweight and obesity and improving overall health.

## Figures and Tables

**Table 1:** Total sugar intake consumed by 6–8 years old children (*n* = 2237) in Valencia, Spain, 2018.

Participants	Total Sugar Intake (g)												
	Mean	Median	SD	IQR	Minimum	Maximum	P5	P10	P25	P50	P75	P90	P95
Total (*n* = 2237)	93.77	91.85	25.72	32.10	24.60	348.00	55.40	63.80	76.90	91.85	109.00	125.00	136.00
Boys (*n* = 1098)	96.24	94.90	24.34	32.10	24.60	250.00	58.28	66.94	79.90	94.90	112.00	127.00	138.00
Girls (*n* = 1139)	91.38	88.80	26.78	31.50	25.10	348.00	53.02	61.30	74.50	88.80	106.00	122.00	133.00

SD: standard deviation; IQR: interquartile range.

**Table 2:** Breakdown of total sugar intake consumed by 6–8 years old children (*n* = 2237) in Valencia, Spain, 2018.

Sugars	Total (*n* = 2237)				Boys (*n* = 1098)				Girls (*n* = 1139)				*p*-Value *^a^
	Mean	SD	Minimum	Maximum	Mean	SD	Minimum	Maximum	Mean	SD	Minimum	Maximum	
Total sugar intake (g)	93.77	25.72	24.60	348.00	96.24	24.34	24.60	250.00	91.38	26.78	25.10	348.00	<0.001
Glucose (g)	8.10	4.40	0.00	43.40	8.46	4.46	0.18	35.10	7.75	4.31	0.00	43.40	<0.001
Fructose (g)	11.04	6.10	0.00	56.30	11.55	6.24	0.14	45.30	10.55	5.93	0.00	56.30	<0.001
Galactose (g)	0.16	0.35	0.00	3.10	0.16	0.34	0.00	2.20	0.16	0.35	0.00	3.10	1.000
Sucrose (g)	22.70	13.33	0.09	276.00	23.30	11.28	0.35	133.00	22.12	15.04	0.09	276.00	0.036
Maltose (g)	0.13	0.25	0.00	2.80	0.13	0.25	0.00	2.40	0.14	0.25	0.00	2.80	0.344
Lactose (g)	14.58	7.29	0.00	68.40	14.94	7.10	0.00	55.60	14.23	7.46	0.00	68.40	0.021

SD: standard deviation. * *p*-Value < 0.05 considered statistically significant. ^a^ Mean and SD of boys versus girls were compared with the ANOVA Test.

**Table 3:** Sociodemographic characteristics of schoolchildren in relation to sugar intake consumed by 6–8 years old children (*n* = 2237) in Valencia, Spain, 2018.

Characteristics	Simple Sugars Intake (93.77 ± 25.72 g)								
	Boys (*n* = 1098)				Girls (*n* = 1139)				
	Simple Sugars Intake (96.24 ± 24.34 g)				Simple Sugars Intake (91.38 ± 26.78 g)				
	P25 (<79.9 g) (*n* = 276)	P25–P75 (79.9–112.0 g) (*n* = 542)	P75 (>112.0 g) (*n* = 280)	*p*-Value *^a^	P25 (<74.5 g)(*n* = 324)	P25–P75 (74.5–106.0 g) (*n* = 526)	P75 (>106.0 g) (*n* = 289)	*p*-Value *^b^	*p*-Value *^c^
	%	%	%		%	%	%		
Fathers’ educational level									
Low	31.4	28.0	20.0	˂0.001	31.5	28.5	20.6	0.017	˂0.001
Average	36.4	34.6	28.5	˂0.001	30.5	33.8	32.1	0.016	˂0.001
High	32.2	37.5	51.5	˂0.001	38.0	37.7	47.3	0.003	0.025
Mothers’ educational level									
Low	18.7	11.1	8.2	˂0.001	17.3	16.8	11.6	0.025	˂0.001
Average	39.3	35.9	26.8	˂0.001	35.0	32.3	27.9	0.023	˂0.001
High	42.1	52.9	65.1	˂0.001	47.7	50.9	60.5	0.003	0.194
Level of physical activity									
Inadequate	14.3	14.6	10.4	0.233	22.3	21.1	23.2	0.778	˂0.001
Adequate	85.7	85.4	89.6	0.233	77.7	78.9	76.8	0.778	˂0.001
Mediterranean diet adherence									
Low	7.5	6.5	4.1	0.172	3.7	5.1	4.4	0.623	0.189
Average	46.3	43.7	42.3	0.594	36.6	43.4	48.4	0.013	0.073
Optimum	46.3	49.8	53.6	0.236	59.8	51.5	47.3	0.006	0.010

SD: standard deviation. Mean and SD were compared with the ANOVA Test. * *p*-Value < 0.05 considered statistically significant. ^a^ Comparison between all boys. ^b^ Comparison between all girls. ^c^ Comparison between all children.

**Table 4:** Sociodemographic characteristics of schoolchildren in relation to % of total energy intake from sugar intake consumed by 6–8 years old children (*n* = 2237) in Valencia, Spain, 2018.

Characteristics	% of Total Energy from Sugar (21.07 ± 12.09%)								
	Boys (*n* = 1098)				Girls (*n* = 1139)				
	% of Total Energy from Sugar (21.15 ± 11.90%)				% of Total Energy from Sugar (20.99 ± 12.26%)				
	P25 (<14.99%) (*n* = 274)	P25–P75 (14.99%–21.17%) (*n* = 549)	P75 (>21.17%) (*n* = 275)	*p*-Value *^a^	P25 (<14.61%) (*n* = 284)	P25–P75 (14.61%–20.90%) (*n* = 570)	P75 (>20.90%) (*n* = 285)	*p*-Value *^b^	*p*-Value *^c^
	%	%	%		%	%	%		
Fathers’ educational level									
Low	32.4	27.0	28.7	0.257	31.9	27.7	28.3	0.411	0.479
Average	34.6	34.2	34.3	0.991	35.1	33.9	34.6	0.918	0.999
High	33.1	38.8	36.9	0.293	33.0	38.4	37.1	0.311	0.442
Mothers’ educational level									
Low	16.7	13.6	15.2	0.480	16.3	14.1	14.4	0.693	0.818
Average	38.0	34.3	34.2	0.533	38.7	34.0	35.1	0.396	0.679
High	45.3	52.1	50.6	0.177	45.0	51.8	50.6	0.176	0.225
Level of physical activity									
Inadequate	17.7	17.3	17.1	0.968	18.3	16.8	18.3	0.700	0.994
Adequate	82.3	82.7	82.9	0.968	81.7	83.2	81.7	0.700	0.994
Mediterranean diet adherence									
Low	4.9	5.6	6.0	0.828	4.7	5.6	6.1	0.742	0.965
Average	45.6	42.4	41.6	0.575	44.9	42.4	43.1	0.767	0.894
Optimum	49.5	52.1	52.4	0.763	50.3	52.0	50.8	0.899	0.979

SD: standard deviation. Mean and SD were compared with the ANOVA Test. * *p*-Value < 0.05 considered statistically significant. ^a^ Comparison between all boys. ^b^ Comparison between all girls. ^c^ Comparison between all children.

**Table 5:** Anthropometric characteristics of schoolchildren in relation to sugar intake consumed by 6–8 years old children (*n* = 2237) in Valencia, Spain, 2018.

Characteristics	Simple Sugars Intake (93.77 ± 25.72 g)														
	Boys (*n* = 1098)							Girls (*n* = 1139)							
	Simple Sugars Intake (96.24 ± 24.34 g)							Simple Sugars Intake (91.38 ± 26.78 g)							
	P25 (<79.9 g) (*n* = 276)		P25–P75 (79.9–112.0 g) (*n* = 542)		P75 (>112.0 g) (*n* = 280)		*p*-Value *^a^	P25 (<74.5 g) (*n* = 324)		P25–P75 (74.5–106.0 g) (*n* = 526)		P75 (>106.0 g) (*n* = 289)		*p*-Value *^b^	p-Value *^c^
	Mean	SD	Mean	SD	Mean	SD		Mean	SD	Mean	SD	Mean	SD		
Age (years)	7.31	1.04	7.24	1.01	7.44	1.03	0.026	7.34	1.03	7.29	1.02	7.32	1.03	0.826	0.178
Weight (kg)	29.15	7.98	28.74	6.92	29.31	7.16	0.518	29.46	7.50	28.81	7.05	28.27	6.95	0.117	0.332
Weight z-score	2.54	11.61	1.15	4.24	0.79	1.08	0.003	1.78	8.64	0.91	2.33	0.77	2.32	0.018	0.001
Height (m)	1.28	0.08	1.28	0.08	1.29	0.08	0.605	1.29	0.08	1.28	0.08	1.27	0.08	0.183	0.306
Height z-score	1.91	9.86	1.05	5.18	0.55	1.01	0.030	1.40	8.06	1.04	5.76	0.75	4.13	0.424	0.120
BMI	17.41	3.20	17.04	2.56	17.25	2.57	0.173	17.58	2.95	17.26	2.85	17.01	2.57	0.042	0.057
BMI z-score	2.27	13.14	0.88	4.10	0.66	1.14	0.013	1.54	7.91	0.68	1.75	0.54	1.41	0.007	0.002
Waist (cm)	60.43	9.65	61.28	6.84	61.02	7.62	0.397	61.41	7.75	60.78	7.13	59.32	7.59	0.002	0.007
Hip (cm)	69.16	10.08	70.78	7.07	70.48	7.83	0.041	70.86	8.20	70.07	8.39	69.28	7.82	0.083	0.039
Waist/hip Index	0.87	0.05	0.86	0.56	1.20	5.48	0.257	0.89	0.46	0.87	0.28	0.85	0.05	0.312	0.272
Fat percentage	16.79	10.74	16.09	9.10	18.43	6.87	0.024	18.55	10.17	16.84	9.43	20.59	7.08	0.001	0.001

SD: standard deviation. Mean and SD were compared with the ANOVA Test. * *p*-Value < 0.05 considered statistically significant. ^a^ Comparison between all boys. ^b^ Comparison between all girls. ^c^ Comparison between all children.

**Table 6:** Anthropometric characteristics of schoolchildren in relation to % of total energy intake from sugar intake consumed by 6–8 years old children (*n* = 2237) in Valencia, Spain, 2018.

Characteristics	% of Total Energy from Sugar (21.07 ± 12.09%)														
	Boys (*n* = 1098)							Girls (*n* = 1139)							
	% of Total Energy from Sugar (21.15 ± 11.90%)							% of Total Energy from Sugar (20.99 ± 12.26%)							
	P25 (<14.99%) (*n* = 274)		P25–P75 (14.99%–21.17%) (*n* = 549)		P75 (>21.17%) (*n* = 275)		*p*-Value *^a^	P25 (<14.61%) (*n* = 284)		P25–P75 (14.61%–20.90%) (*n* = 570)		P75 (>20.90%) (*n* = 285)		*p*-Value *^b^	*p*-Value *^c^
	Mean	SD	Mean	SD	Mean	SD		Mean	SD	Mean	SD	Mean	SD		
Weight (kg)	29.37	8.14	29.03	7.01	28.55	6.83	0.413	29.69	7.25	28.76	7.15	28.25	7.08	0.050	0.163
Weight z-score	2.17	10.50	1.33	5.54	0.81	1.16	0.049	1.85	9.24	0.91	2.25	0.82	2.36	0.020	0.010
Height (m)	1.30	0.09	1.29	0.08	1.28	0.08	0.242	1.30	0.08	1.29	0.08	1.27	0.09	0.005	˂0.001
Height z-score	1.80	9.75	1.08	5.29	0.59	1.10	0.067	1.88	10.20	0.83	1.71	0.64	1.46	0.038	0.016
BMI	17.24	3.05	17.21	2.70	17.10	2.49	0.791	17.39	2.84	17.29	2.86	17.21	2.75	0.755	0.877
BMI z-score	1.85	11.88	1.11	5.77	0.64	1.20	0.141	1.56	8.45	0.68	1.71	0.64	1.46	0.014	0.037
Waist (cm)	60.95	9.65	61.65	7.07	59.76	6.92	0.010	61.54	7.64	60.91	7.29	58.91	7.39	0.009	˂0.001
Hip (cm)	69.78	10.07	70.83	7.22	69.82	7.51	0.145	71.08	8.14	70.30	7.98	68.64	8.56	0.003	0.002
Waist/hip Index	0.87	0.06	0.87	0.06	0.96	2.81	0.232	0.90	0.48	0.87	0.05	0.88	0.31	0.430	0.862
Fat percentage	16.17	10.46	16.00	9.02	19.02	6.78	˂0.001	18.13	9.85	16.79	9.67	21.59	6.20	˂0.001	˂0.001

SD: standard deviation. Mean and SD were compared with the ANOVA Test. * *p*-Value < 0.05 considered statistically significant. ^a^ Comparison between all boys. ^b^ Comparison between all girls. ^c^ Comparison between all children.

**Table 7:** Results of the adjusted multiple regression analysis of the possible association between anthropometric variables and the percentage of total energy derived from total sugar intake by 6–8 years old children (*n* = 2237) in Valencia, Spain, 2018.

Characteristics	% of Total Energy from Sugar (21.07% ± 12.09%)											
	Boys (*n* = 1098)						Girls (*n* = 1139)					
	% of Total Energy from Sugar (21.15% ± 11.90%)						% of Total Energy from Sugar (20.99% ± 12.26%)					
	P25 (<14.99%) (*n* = 274)		P25–P75 (14.995–21.17%) (*n* = 549)		P75 (>21.17%) (*n* = 275)		P25 (<14.61%) (*n* = 284)		P25–P75 (14.61%–20.90%) (*n* = 570)		P75 (>20.90%) (*n* = 285)	
	*R* ^2^	*p*-Value	*R* ^2^	*p*-Value	*R* ^2^	*p*-Value	*R* ^2^	*p*-Value	*R* ^2^	*p*-Value	*R* ^2^	*p*-Value
Weight (kg)	0.298	0.001	0.303	0.001	0.322	0.001	0.270	0.001	0.308	0.001	0.312	0.001
Weight z-score	0.004	0.664	0.004	0.959	0.065	0.007	0.006	0.462	0.003	0.919	0.001	0.421
Height (m)	0.331	0.001	0.328	0.001	0.401	0.001	0.283	0.001	0.324	0.001	0.399	0.001
Height z-score	0.006	0.818	0.001	0.366	0.003	0.317	0.003	0.232	0.002	0.643	0.001	0.289
BMI	0.079	0.001	0.084	0.001	0.099	0.001	0.055	0.001	0.089	0.001	0.094	0.001
BMI z-score	0.004	0.556	0.004	0.939	0.113	0.001	0.004	0.640	0.001	0.283	0.009	0.687
Waist (cm)	0.114	0.113	0.129	0.001	0.118	0.001	0.095	0.001	0.131	0.001	0.123	0.001
Hip (cm)	0.001	0.701	0.219	0.001	0.173	0.001	0.156	0.001	0.213	0.001	0.182	0.001
Waist/hip Index	0.016	0.014	0.020	0.001	0.001	0.716	0.007	0.184	0.015	0.001	0.001	0.713
Fat percentage	0.001	0.404	0.001	0.748	0.006	0.202	0.003	0.468	0.001	0.466	0.003	0.405

* *p*-value < 0.05 considered statistically significant.

**Table 8:** Macronutrients intake consumed by 6–8-years old children (*n* = 2237) in Valencia, Spain, 2018.

	Simple Sugars Intake (93.77 ± 25.72 g)														
Macronutrient RDI	Boys (*n* = 1098)							Girls (*n* = 1139)							
	Simple Sugars Intake (96.24 ± 24.34 g)							Simple Sugars Intake (91.38 ± 26.78 g)							
	P25 (<79.9 g) (*n* = 276)		P25–P75 (79.9–112.0 g) (*n* = 542)		P75 (>112.0 g) (*n* = 280)		*p*-value *^a^	P25 (<74.5 g) (*n* = 324)		P25–P75 (74.5–106.0 g) (*n* = 526)		P75 (>106.0 g) (*n* = 289)		*p*-Value *^b^	*p*-Value *^c^
	Mean	SD	Mean	SD	Mean	SD		Mean	SD	Mean	SD	Mean	SD		
Energy 1500–1700 kcal	2004.67	345.14	2270.34	356.60	2630.55	663.38	˂0.001	1939.44	328.49	2197.29	359.56	2540.01	609.11	˂0.001	˂0.001
Protein 19 g	86.96	21.23	93.35	18.85	104.04	38.91	˂0.001	82.99	21.12	89.54	18.41	98.12	24.14	˂0.001	˂0.001
Carbohydrate 130 g	202.13	43.85	233.57	35.58	281.32	54.39	˂0.001	191.19	36.83	222.81	35.02	269.47	55.52	˂0.001	˂0.001
Glucose (g)	5.95	2.89	8.76	4.06	11.85	5.41	˂0.001	5.41	2.68	8.09	3.47	11.40	6.20	˂0.001	˂0.001
Fructose (g)	8.34	4.33	11.98	5.88	15.69	7.32	˂0.001	7.48	3.94	11.05	5.02	15.11	8.20	˂0.001	˂0.001
Galactose (g)	0.05	0.18	0.19	0.37	0.23	0.42	˂0.001	0.08	0.24	0.17	0.36	0.28	0.49	˂0.001	˂0.001
Lactose (g)	11.96	6.06	15.47	6.27	18.32	9.24	˂0.001	11.59	6.28	14.64	6.65	18.24	9.80	˂0.001	˂0.001
Maltose (g)	0.08	0.16	0.15	0.27	0.18	0.30	˂0.001	0.08	0.17	0.15	0.25	0.21	0.37	˂0.001	˂0.001
Sucrose (g)	15.82	6.86	24.16	8.98	33.36	15.05	˂0.001	14.52	7.01	23.04	8.89	34.47	28.21	˂0.001	˂0.001
Fiber 25 g	16.96	5.67	19.34	8.17	21.23	7.03	˂0.001	15.86	4.55	18.79	5.28	21.65	13.41	˂0.001	˂0.001
Cholesterol (mg)	327.18	102.53	353.77	101.43	392.19	259.85	˂0.001	304.94	85.77	344.10	106.30	369.79	117.50	˂0.001	˂0.001
Lipid (g)	92.88	21.82	103.42	23.00	116.51	40.17	˂0.001	91.37	21.22	101.45	23.49	114.14	37.72	˂0.001	˂0.001
SFA (g)	30.71	7.24	34.72	7.68	38.92	11.12	˂0.001	29.38	6.89	33.89	7.74	39.17	11.87	˂0.001	˂0.001
Myristic Acid (g)	2.31	0.83	2.66	0.89	2.92	1.09	˂0.001	2.15	0.81	2.57	0.86	3.01	1.68	˂0.001	˂0.001
Palmitic Acid (g)	16.59	3.99	18.42	4.37	20.38	6.71	˂0.001	15.90	3.79	17.90	4.33	20.34	5.89	˂0.001	˂0.001
Stearic Acid (g)	6.83	1.78	7.66	1.99	8.50	2.78	˂0.001	6.58	1.78	7.56	1.99	8.65	2.48	˂0.001	˂0.001
MUFA (g)	40.88	11.44	45.42	12.18	52.32	23.81	˂0.001	40.25	11.67	44.61	12.42	50.85	22.83	˂0.001	˂0.001
Palmitoleic Acid (g)	1.51	0.40	1.66	0.43	1.74	0.59	˂0.001	1.45	0.40	1.60	0.45	1.72	0.56	˂0.001	˂0.001
Oleic Acid (g)	37.25	10.71	41.65	11.38	47.98	22.75	˂0.001	37.09	11.03	40.89	11.71	46.88	22.18	˂0.001	˂0.001
PUFA (g)	11.72	3.39	12.95	4.08	14.14	4.69	˂0.001	11.44	3.46	12.71	4.38	13.76	4.85	˂0.001	˂0.001
ω-6 PUFA 10 g	7.00	4.38	8.66	4.53	8.90	5.01	˂0.001	7.68	4.00	8.20	4.67	8.87	5.49	˂0.001	˂0.001
ω-3 PUFA 0.9 g	0.94	0.73	1.31	0.84	1.26	0.92	˂0.001	1.09	0.71	1.20	0.86	1.21	0.94	˂0.001	˂0.001
Linoleic Acid 10 g	9.41	2.97	10.37	3.40	11.35	3.73	˂0.001	9.28	3.01	10.24	3.75	11.16	4.06	˂0.001	˂0.001
Linolenic Acid 0.9 g	1.03	0.35	1.22	0.64	1.31	0.57	˂0.001	1.03	0.35	1.18	0.54	1.30	0.65	˂0.001	˂0.001
Arachidonic Acid (g)	0.17	0.09	0.18	0.10	0.16	0.09	˂0.001	0.15	0.08	0.17	0.09	0.17	0.10	˂0.001	˂0.001

SD: standard deviation; SFA: saturated fatty acid; MUFA: monounsaturated fatty acid; PUFA: polyunsaturated fatty acid. Mean and SD were compared with the ANOVA Test. * *p*-Value < 0.05 considered statistically significant. ^a^ Comparison between all boys. ^b^ Comparison between all girls. ^c^ Comparison between all children.

**Table 9:** Macronutrients intake consumed by percentage of total energy derived from total sugar intake by 6–8 years old children (*n* = 2237) in Valencia, Spain, 2018.

	% of Total Energy from Sugar (21.07 ± 12.09%)														
Macronutrient	Boys (*n* = 1098)							Girls (*n* = 1139)							
	% of Total Energy from Sugar (21.15% ± 11.90%)							% of Total Energy from Sugar (20.99% ± 12.26%)							
	P25 (<14.99%) (*n* = 274)		P25–P75 (14.99%–21.17%) (*n* = 549)		P75 (>21.17%) (*n* = 275)		*p*-Value *^a^	P25 (<14.61%) (*n* = 284)		P25–P75 (14.61%–20.90%) (*n* = 570)		P75 (>20.90%) (*n* = 285)		*p*-Value *^b^	*p*-Value *^c^
	Mean	SD	Mean	SD	Mean	SD		Mean	SD	Mean	SD	Mean	SD		
Energy (kcal)	2004.67	345.14	2270.34	356.60	2236.68	716.33	˂0.001	1939.44	328.49	2197.29	359.56	2168.21	672.69	˂ 0.001	˂0.001
Protein	16.91	2.87	16.52	2.40	15.72	2.37	˂0.001	16.98	2.97	16.51	2.38	16.52	2.57	˂0.001	˂0.001
Carbohydrate	38.72	5.49	41.43	4.64	44.91	4.22	˂0.001	38.50	5.64	41.32	4.62	44.73	4.22	˂0.001	˂0.001
Glucose	1.05	0.53	1.53	0.66	2.15	1.06	˂0.001	1.02	0.51	1.51	0.65	2.13	1.04	˂0.001	˂0.001
Fructose	1.47	0.77	2.10	0.98	2.83	1.41	˂0.001	1.44	0.75	2.06	0.97	2.81	1.39	˂0.001	˂0.001
Galactose	0.01	0.04	0.03	0.06	0.06	0.10	˂0.001	0.01	0.04	0.03	0.06	0.06	0.09	˂0.001	˂0.001
Lactose	2.21	1.18	2.73	1.20	2.50	1.62	˂0.001	2.17	1.16	2.71	1.21	3.45	1.59	˂0.001	˂0.001
Maltose	0.01	0.03	0.03	0.04	0.03	0.06	˂0.001	0.01	0.03	0.02	0.04	0.03	0.06	˂0.001	˂0.001
Sucrose	2.83	1.24	4.31	1.61	5.99	3.02	˂0.001	2.73	1.19	4.24	1.59	5.94	2.94	˂0.001	˂0.001
Fiber (g)	8.08	2.12	8.54	2.74	8.24	1.95	˂0.001	8.07	2.16	8.51	2.72	8.29	1.94	0.003	˂0.001
Cholesterol (mg)	159.79	44.50	156.24	37.58	146.76	40.45	˂0.001	160.75	45.30	155.84	37.28	148.18	41.38	˂0.001	˂0.001
Lipid (g)	48.14	4.78	45.18	4.47	41.88	4.66	˂0.001	48.38	4.84	45.31	4.44	41.97	4.64	˂0.001	˂0.001
SFA (g)	15.45	2.38	15.21	2.19	14.95	2.38	0.007	15.49	2.41	15.22	2.20	14.94	2.33	0.003	0.011
Myristic Acid (g)	1.14	0.41	1.16	0.35	1.17	0.38	0.360	1.14	0.41	1.15	0.35	1.17	0.37	0.445	0.844
Palmitic Acid (g)	8.33	1.27	8.08	1.22	7.63	1.31	0.001	8.36	1.27	8.09	1.22	7.64	1.29	˂0.001	˂0.001
Stearic Acid (g)	3.42	0.63	3.38	0.64	3.30	0.73	0.038	3.44	0.64	3.37	0.64	3.30	0.71	0.010	0.047
MUFA (g)	21.43	3.95	19.80	3.15	18.09	2.98	˂0.001	21.57	4.00	19.87	3.16	18.12	3.00	˂0.001	˂0.001
Palmitoleic Acid (g)	0.75	0.15	0.72	0.14	0.67	0.14	˂0.001	0.76	0.15	0.72	0.14	0.67	0.14	˂0.001	˂0.001
Oleic Acid (g)	19.63	3.94	18.17	3.12	16.61	2.97	˂0.001	19.76	3.99	18.23	1.13	16.64	2.99	˂0.001	˂0.001
PUFA (g)	6.03	1.42	5.65	1.7	5.04	1.02	˂0.001	6.07	1.41	5.67	1.20	5.05	1.00	˂0.001	˂0.001
ω-6 PUFA (g)	3.87	2.01	3.71	1.75	3.11	1.63	˂0.001	3.91	2.03	3.71	1.76	3.13	1.62	˂0.001	˂0.001
ω-3 PUFA (g)	0.53	0.36	0.54	0.34	0.46	0.35	0.002	0.53	0.37	0.54	0.34	0.47	0.34	0.003	0.003
Linoleic Acid (g)	4.86	1.25	4.54	1.05	4.06	0.90	˂0.001	4.91	1.26	4.55	1.07	4.07	0.88	˂0.001	˂0.001
Linolenic Acid (g)	0.54	0.22	0.52	0.15	0.48	0.16	˂0.001	0.55	0.23	0.52	0.15	0.48	0.16	˂0.001	˂0.001
Arachidonic Acid (g)	0.08	0.04	0.07	0.03	0.07	0.03	˂0.001	0.08	0.04	0.07	0.04	0.07	0.03	˂0.001	˂0.001

Values are presented as % of total energy for protein, carbohydrate, glucose, fructose, galactose, lactose, maltose, and sucrose and as unit/1000 kcal for fiber, cholesterol, lipid, SFA, myristic acid, palmitic acid, stearic acid, MUFA, palmitoleic acid, oleic acid, PUFA, ω-6 PUFA, ω-3 PUFA, linoleic acid, linolenic acid, and arachidonic acid. SD: standard deviation; SFA: saturated fatty acid; MUFA: monounsaturated fatty acid; PUFA: polyunsaturated fatty acid. Mean and SD were compared with the ANOVA Test. * *p*-Value < 0.05 considered statistically significant. ^a^ Comparison between all boys. ^b^ Comparison between all girls. ^c^ Comparison between all children.

**Table 10:** Micronutrients intake consumed by 6–8 years old children (*n* = 2237) in Valencia, Spain, 2018.

Micronutrient RDI	Simple Sugars Intake (93.77 ± 25.72 g)														
	Boys (*n* = 1098)							Girls (*n* = 1139)							
	Simple Sugars Intake (96.24 ± 24.34 g)							Simple Sugars Intake (91.38 ± 26.78 g)							
	P25 (<79.9 g) (*n* = 276)		P25–P75 (79.9–112.0 g) (*n* = 542)		P75 (>112.0 g) (*n* = 280)		*p*-Value *^a^	P25 (<74.5 g) (*n* = 324)		P25–P75 (74.5–106.0 g) (*n* = 526)		P75 (>106.0 g) (*n* = 289)		*p*-Value *^b^	*p*-Value *^c^
	Mean	SD	Mean	SD	Mean	SD		Mean	SD	Mean	SD	Mean	SD		
Mineral RDI															
Ca 800 mg	832.20	201.39	1010.05	219.31	1062.73	311.12	˂0.001	797.50	208.97	967.86	215.00	1156.60	327.88	˂0.001	˂0.001
Fe 9 mg	12.24	3.23	13.46	3.87	15.80	7.84	˂0.001	11.62	3.46	12.93	3.54	14.88	6.01	˂0.001	˂0.001
I 120µg	89.50	27.07	105.72	62.20	123.57	113.06	˂0.001	86.14	26.75	98.74	27.51	113.75	38.58	˂0.001	˂0.001
Mg 170 mg	259.14	65.34	300.21	64.44	349.33	122.89	˂0.001	247.57	56.44	287.14	55.19	336.08	106.74	˂0.001	˂0.001
Zinc 6.5 mg	8.95	1.95	9.92	2.08	11.07	3.90	˂0.001	8.52	1.92	9.56	2.02	10.65	2.80	˂0.001	˂0.001
Vitamin RDI															
A 450 µg	866.14	1051.02	985.54	571.89	1099.67	757.48	˂0.001	832.38	1045.39	947.15	544.91	1185.26	1289.60	˂0.001	˂0.001
B1 0.8 mg	1.67	2.54	1.61	1.32	1.74	1.18	0.672	1.48	1.78	1.46	0.64	1.66	0.80	0.918	0.213
B2 1.1 mg	1.67	0.42	1.95	0.41	2.29	0.71	˂0.001	1.60	0.47	1.90	0.46	2.20	0.70	˂0.001	˂0.001
B6 1.0 mg	1.90	0.51	2.17	0.59	2.45	1.14	˂0.001	1.80	0.56	2.08	0.58	2.34	0.99	˂0.001	˂0.001
Folate 200 µg	7.87	15.40	10.24	17.86	16.56	37.81	0.247	8.98	19.13	10.68	19.91	13.30	27.23	0.235	0.337
B12 1.2 µg	5.67	4.74	5.95	2.83	6.96	5.08	˂0.001	5.15	3.02	5.80	2.64	6.19	2.71	˂0.001	˂0.001
C 45 mg	78.48	35.93	104.77	49.33	129.95	59.72	˂0.001	77.53	36.94	99.95	42.05	131.08	85.81	˂0.001	˂0.001
D 5 µg	2.84	2.94	3.03	2.36	3.51	3.45	˂0.001	2.56	2.39	2.90	2.24	3.44	2.86	˂0.001	˂0.001
E 7.0 mg	6.96	2.53	8.12	3.17	9.55	3.95	0.017	6.68	2.36	7.93	2.85	3.33	4.34	˂0.001	˂0.001

SD: standard deviation. Mean and SD were compared with the ANOVA Test. * *p*-Value < 0.05 considered statistically significant. ^a^ Comparison between all boys. ^b^ Comparison between all girls. ^c^ Comparison between all children.

**Table 11:** Micronutrient intake consumed by percentage of total energy derived from total sugar intake by 6–8 years old children (*n* = 2237) in Valencia, Spain, 2018.

	% of Total Energy from Sugar (21.07 ± 12.09%)														
Micronutrient	Boys (*n* = 1098)							Girls (*n* = 1139)							
	% of Total Energy from Sugar (21.15% ± 11.90%)							% of Total Energy from Sugar (20.99% ± 12.26%)							
	P25 (<14.99%) (*n* = 274)		P25–P75 (14.99%–21.17%) (*n* = 549)		P75 (>21.17%) (*n* = 275)		*p*-Value *^a^	P25 (<14.61%) (*n* = 284)		P25–P75 (14.61%–20.90%) (*n* = 570)		P75 (>20.90%) (*n* = 285)		*p*-Value *^b^	*p*-Value *^c^
	Mean	SD	Mean	SD	Mean	SD		Mean	SD	Mean	SD	Mean	SD		
Mineral															
Ca (mg)	395.48	91.70	449.65	97.23	498.00	115.29	˂0.001	393.19	91.09	446.32	97.19	498.01	114.10	˂0.001	˂0.001
Fe (mg)	5.91	1.34	6.04	1.54	5.73	1.47	0.004	5.93	1.38	6.02	1.53	5.76	1.44	0.018	0.015
I (µg)	43.58	14.96	46.27	19.83	47.31	12.40	0.002	43.57	15.39	46.03	19.56	47.45	12.38	0.003	0.011
Mg (mg)	124.96	24.90	133.51	21.67	136.45	23.08	˂0.001	125.26	25.67	132.72	21.53	136.57	23.24	˂0.001	˂0.001
Zinc (mg)	4.34	0.75	4.39	0.73	4.35	0.73	0.364	4.36	0.76	4.37	0.73	4.36	0.73	0.898	0.952
Vitamin															
A (µg)	430.90	485.35	432.28	250.20	433.51	333.26	0.993	435.03	513.31	428.63	246.95	439.16	202.50	0.856	0.998
B1 (mg)	0.74	0.87	0.71	0.67	0.65	0.17	0.173	0.75	0.91	0.71	0.67	0.65	0.17	0.123	0.297
B2 (mg)	0.79	0.18	0.87	0.19	0.93	0.21	˂0.001	0.79	0.18	0.86	0.19	0.93	0.21	˂0.001	˂0.001
B6 (mg)	0.91	0.22	0.96	0.24	0.93	0.26	˂0.001	0.91	0.22	0.96	0.21	0.94	0.25	˂0.001	˂0.001
Folate (µg)	3.91	7.63	5.08	9.76	5.44	10.13	0.018	3.93	7.71	5.01	9.67	5.38	9.93	0.038	0.153
B12 (µg)	2.69	1.73	2.67	1.23	2.46	0.81	0.038	2.71	1.82	2.66	1.22	2.49	0.82	0.051	0.065
C (mg)	37.79	18.38	46.97	20.48	54.19	24.43	˂0.001	37.36	18.08	46.43	20.35	54.12	24.51	˂0.001	˂0.001
D (µg)	1.32	1.20	1.38	1.13	1.22	0.96	0.097	1.30	1.21	1.38	1.13	1.25	0.96	0.132	0.248
E (mg)	3.57	1.15	3.56	1.05	3.42	1.00	0.107	3.56	1.14	3.56	1.07	3.44	0.99	0.184	0.264

Values are presented as unit/1000 kcal. SD: standard deviation. Mean and SD were compared with the ANOVA Test. * *p*-Value < 0.05 considered statistically significant. ^a^ Comparison between all boys. ^b^ Comparison between all girls. ^c^ Comparison between all children.
